# Self‐Powered Photodetection and Optical Information Recognition in a Mixed‐Dimensional WSe_2_/CdSe Heterojunction

**DOI:** 10.1002/nap2.70362

**Published:** 2026-11-07

**Authors:** Lei Liu, Haoyun Dou, Haiqiang Huang, Fan Mu, Yan Yang, Hui Zhang, Feng Song, Yingkai Liu

**Affiliations:** ^1^ Institute of Physics and Electronic Information Yunnan Normal University Kunming China; ^2^ Yunnan Key Laboratory of Opto‐electronic Information Technology Yunnan Normal University Kunming China; ^3^ Southwest United Graduate School Kunming China; ^4^ College of Physics Nankai University Tianjin China

**Keywords:** interfacial electrostatics, optical information recognition, photocurrent mapping, self‐powered photodetection, WSe_2_/CdSe mixed‐dimensional heterojunctions

## Abstract

Mixed‐dimensional van der Waals heterojunctions offer opportunities for self‐powered optical sensing and information processing, yet identifying the spatial origin of zero‐bias photoresponse remains important for understanding device operation. Here, a vertical WSe_2_/CdSe heterojunction composed of a WSe_2_ nanosheet and a CdSe nanobelt is investigated for visible photodetection. Under 610 nm illumination, the device exhibits a photovoltaic response with an open‐circuit voltage of 0.403 V and a short‐circuit current of 4.77 × 10^−10^ A. Quantitative photocurrent mapping reveals a mean response of 5.94 nA in the heterojunction overlap region, approximately 50 times that near the metal contacts. Scanning Kelvin probe microscopy yields a reproducible contact‐potential‐difference contrast of 293 ± 15 mV, whereas electronic‐structure calculations support a type‐II band‐edge configuration and interfacial charge redistribution. At 100 Hz, the measured rise/fall times are 1.09/0.99 ms at 0 V and 165/191 μs at +3 V. Under +3 V bias, approximately 97% of the reference modulation amplitude is retained at 1 kHz. Self‐powered optical imaging and wavelength‐ and time‐window‐dependent 8‐bit information recognition are further demonstrated, with all 96 tested bits correctly decoded. These results highlight the potential of mixed‐dimensional WSe_2_/CdSe heterojunctions for self‐powered visible‐light sensing and optical information recognition.

## Graphical abstract

A mixed‐dimensional WSe_2_/CdSe heterojunction enables visible‐light photodetection and optical information recognition without external bias. Spatial photocurrent mapping identifies the semiconductor overlap as the principal photoactive region. Wavelength‐dependent photocurrent levels and predefined time windows enable key/payload‐assisted 8‐bit decoding, linking self‐powered sensing with selective optical information recognition.

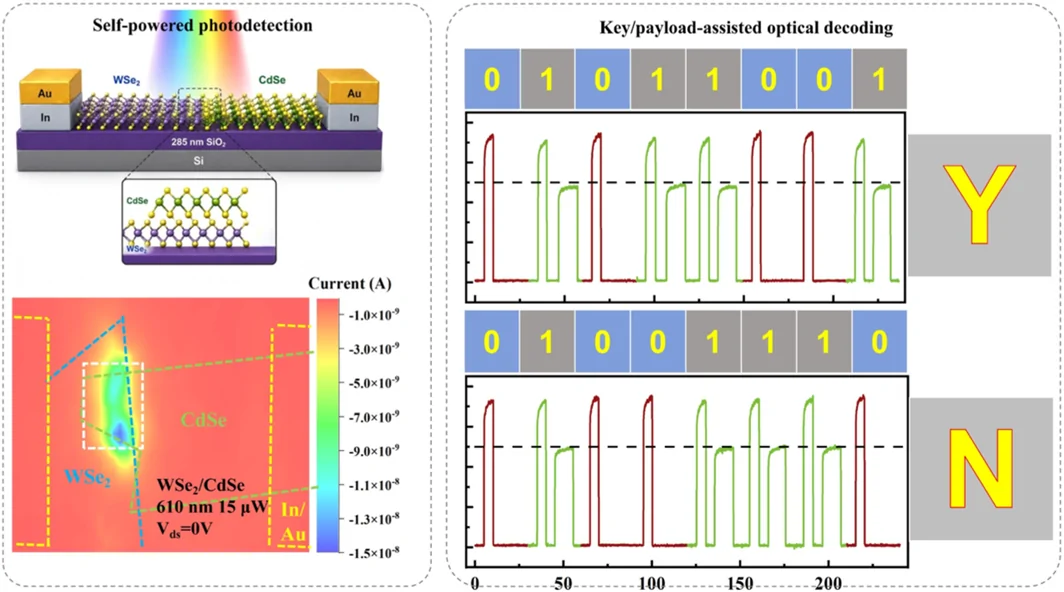

## 1 Introduction

Two‐dimensional transition‐metal dichalcogenides (TMDCs) have attracted considerable interest for optoelectronic devices owing to their thickness‐dependent electronic structures and strong light–matter interactions [1, 2]. However, the limited absorption thickness of atomically thin semiconductors can restrict photocarrier generation, whereas efficient carrier collection without an applied bias requires a suitable internal driving force. Integrating different low‐dimensional semiconductors into van der Waals (vdW) heterojunctions provides a way to regulate interfacial electronic states and promote the separation of photogenerated carriers [3, 4]. Therefore, such heterojunctions are promising for self‐powered photodetection, particularly in optical sensing and imaging systems where low power consumption is desirable [5, 6, 7, 8, 9].

The weak lattice‐matching constraints of vdW integration enable the combination of semiconductors with different electronic structures and photoresponse characteristics [10, 11, 12, 13]. Previous studies have demonstrated that band alignment, interface engineering, and hybrid integration can influence carrier separation, dark current, and spectral response in low‐dimensional heterojunction photodetectors [14, 15, 16, 17, 18, 19, 20, 21]. Nevertheless, the measured photovoltaic output may reflect contributions from different regions of a device, including semiconductor heterointerfaces and metal–semiconductor contacts. Interfacial potential redistribution and localized electronic states can also affect charge separation and transport [22, 23, 24, 25]. Accordingly, spatially resolved photocurrent measurements, combined with electrical and interfacial characterization, are useful for identifying the principal photoactive region and examining the origin of zero‐bias photocurrent.

Representative studies on vdW heterojunction photodetectors have demonstrated that interface design can effectively regulate photovoltaic and optoelectronic properties. For example, MoTe_2_/MoS_2_ vertical heterojunctions have been demonstrated for self‐powered photovoltaic detection [26], whereas WSe_2_/WS_2_ vdW heterojunctions exhibited tunable photovoltaic responses through modulation of interfacial band alignment and carrier transport [27]. WSe_2_/ReS_2_ heterojunctions have further demonstrated the importance of heterointerface electronic structure and spatial carrier generation for photodetection and photovoltaic operation [28]. Beyond conventional photodetection, heterojunction devices have also begun to integrate optical sensing with information‐oriented functions, including wavelength‐sensitive encrypted optical communication and in‐sensor perceptual processing [29, 30, 31, 32]. These developments motivate the integration of photovoltaic detection with optical information recognition using distinguishable optical inputs.

Among various WSe_2_‐based vdW heterostructures, WSe_2_/CdSe is a representative mixed‐dimensional system combining thin WSe_2_ nanosheets and one‐dimensional CdSe nanostructures. Zhang et al. previously demonstrated type‐II interlayer coupling and enhanced bias‐driven photoconductive response in monolayer‐WSe_2_/CdSe nanobelt heterostructures, with the enhancement attributed to interfacial charge transfer and photogating effects [33]. Their work primarily examined the enhancement of photoconductive transport under applied bias. The zero‐bias photovoltaic characteristics of this material combination, particularly the relative contributions of the semiconductor overlap and metal‐contact regions, warrant further investigation. Moreover, the use of its wavelength‐dependent photovoltaic response for optical information recognition remains to be explored.

In this work, we investigate the self‐powered photodetection and optical information recognition capabilities of a vertical WSe_2_/CdSe mixed‐dimensional heterojunction. Particular attention is paid to distinguishing the contributions of the semiconductor overlap and metal‐contact regions to the zero‐bias photoresponse. To this end, spatial photocurrent mapping is combined with photovoltaic measurements, SKPM characterization, and electronic‐structure calculations to examine the relationship between device geometry, interfacial electrostatics, and photovoltaic operation. The bias‐dependent transient and frequency responses are further investigated to evaluate the dynamic characteristics under different operating conditions. Finally, optical imaging and wavelength‐ and time‐window‐dependent information recognition are demonstrated under zero‐bias operation. By examining the spatial origin of photovoltaic response and extending the device functionality to optical information recognition, this study broadens the investigation of mixed‐dimensional WSe_2_/CdSe heterojunctions beyond the previously reported bias‐driven photoconductive behavior.

## 2 Results and Discussion

### 2.1 Device Architecture and Interfacial Characterization of the Vertical WSe_2_/CdSe Heterojunction

Figure 1 illustrates the device architecture and structural characteristics of the vertical WSe_2_/CdSe vdW heterojunction. As shown in Figure 1a, the WSe_2_ nanosheet and CdSe nanobelt form a well‐defined overlap region between the two electrodes. The red dashed square marks the region selected for AFM and SKPM measurements. The inset cross‐sectional schematic illustrates the vertical stacking geometry. The AFM profile along the blue dashed line in Figure 1b gives thicknesses of approximately 1.04 nm for WSe_2_ and 144 nm for the CdSe nanobelt, verifying the nanoscale thickness difference between the two components.

**FIGURE 1:**
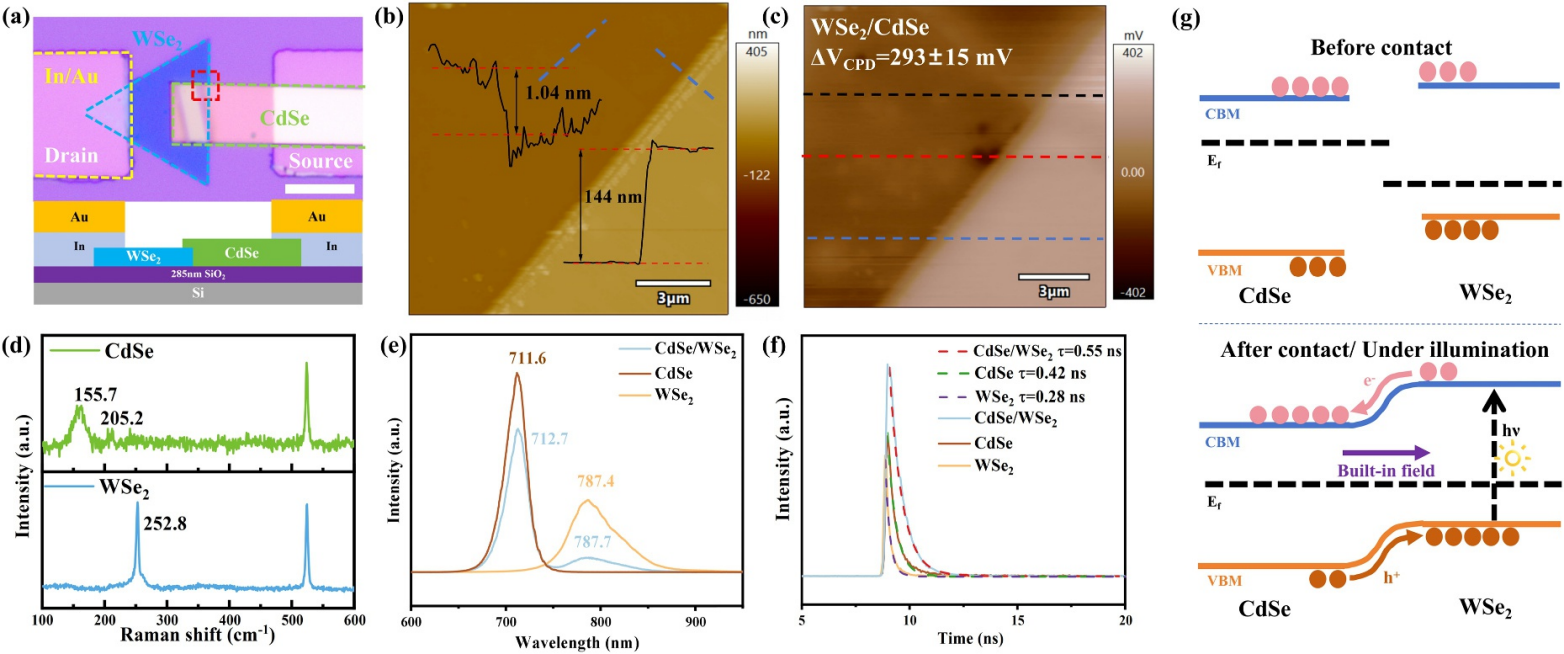
Device architecture and interfacial characterization of the vertical WSe_2_/CdSe heterojunction. (a) Optical image of the device; the red dashed square marks the AFM/SKPM scanning region. Inset: cross‐sectional schematic of the vertical heterojunction. (b) AFM image and corresponding height profile measured along the blue dashed line. (c) SKPM surface‐potential map of the WSe_2_/CdSe interface. The black, red, and blue dashed lines indicate three independent line‐scan positions, yielding an average contact potential difference contrast of ΔV_CPD_ = 293 ± 15 mV. The corresponding profiles are provided in Supporting Information S1: Figure S1. (d) Raman spectra of WSe_2_ and CdSe measured under 532 nm laser excitation. (e) Steady‐state PL spectra of WSe_2_, CdSe, and the WSe_2_/CdSe overlap region measured under 532 nm laser excitation. (f) TRPL decay curves and fitted lifetimes of WSe_2_, CdSe, and the overlap region measured under 375 nm pulsed laser excitation. (g) Qualitative schematic of the proposed band alignment and interfacial carrier separation at the WSe_2_/CdSe heterojunction under illumination.

The interfacial electrostatic contrast was examined by scanning Kelvin probe microscopy (SKPM). As shown in Figure 1c, a clear surface‐potential variation is observed across the WSe_2_/CdSe interface. Three independent line profiles taken across the same interfacial region yield contact‐potential‐difference contrasts of approximately 281, 287, and 309 mV, corresponding to an average value of 293 ± 15 mV. The corresponding line profiles are provided in Supporting Information S1: Figure S1. This reproducible potential contrast indicates substantial interfacial electrostatic redistribution after formation of the heterojunction.

Based on the reported p‐type behavior of WSe_2_ and n‐type transport of CdSe nanobelts [33], together with the electronic‐structure calculations discussed below, Figure 1g presents a qualitative type‐II band‐alignment model. Fermi‐level equilibration upon contact produces interfacial charge redistribution, whereas the band configuration favors spatial separation of photogenerated electrons and holes under illumination. The reproducible SKPM contrast characterizes the accompanying surface electrostatic asymmetry. These results provide an interfacial electronic framework for interpreting the photovoltaic output and spatial photocurrent distribution discussed below.

Raman characterization was then used to verify the vibrational fingerprints of the constituent materials. As shown in Figure 1d, the WSe_2_ nanosheet exhibits a Raman feature at approximately 252.8 cm^−1^, consistent with the overlapping characteristic Raman modes of WSe_2_ [33]. The CdSe nanobelt shows CdSe‐related vibrational features at approximately 155.7 and 205.2 cm^−1^, among which the 205.2 cm^−1^ peak agrees well with the reported LO phonon mode of CdSe [34]. The sharp signal near 520 cm^−1^ is attributed to the Si substrate. These Raman features verify the characteristic vibrational modes of the two components and support the successful integration of WSe_2_ and CdSe into the heterojunction. Steady‐state PL and TRPL measurements were performed to examine the emission and carrier‐relaxation dynamics following heterojunction formation. As shown in Figure 1e, the WSe_2_/CdSe overlap region exhibits substantial PL quenching compared with the exposed constituent regions [14, 22]. Single‐exponential fits to the TRPL decay curves in Figure 1f yield characteristic lifetimes of approximately 0.28 ns for WSe_2_, 0.42 ns for CdSe, and 0.55 ns for the overlap region. The weaker PL and longer fitted lifetime indicate that heterojunction formation modifies both the emission efficiency and decay dynamics. These observations are compatible with reduced radiative recombination associated with spatial carrier separation, whereas the measured decay can also include nonradiative and trap‐related contributions [35, 36]. Together with the SKPM, photovoltaic, and spatial photocurrent results discussed below, the optical measurements support an interface‐associated carrier‐separation picture. The crystalline quality of the WSe_2_ nanosheet and CdSe nanobelt was further examined by TEM, HRTEM, and SAED (Supporting Information S1: Figure S2). The clear lattice fringes and well‐defined diffraction spots confirm the good crystallinity of both materials before heterojunction assembly. Together, the Raman, AFM, SKPM, PL, TRPL, and TEM results establish a structurally defined WSe_2_/CdSe heterojunction with interfacial electronic modulation, providing the basis for investigating self‐powered photodetection.

### 2.2 Zero‐Bias Self‐Powered Photoresponse of the WSe_2_/CdSe Photodetector

Following the structural and interfacial characterization of the vertical heterojunction, the electrical transport and zero‐bias photoresponse characteristics were investigated. As shown in Figure 2a, the dark current remains close to the instrumental detection floor near zero bias, providing a low‐dark‐current baseline for evaluating the self‐powered photoresponse [37, 38]. With increasing bias, the current displays a pronounced polarity‐dependent asymmetry and rises much more rapidly under positive bias than under negative bias, indicating rectifying transport behavior. The zero‐bias spectral responsivity was first measured to identify representative visible wavelengths for subsequent power‐dependent photoresponse measurements. As shown in Figure 2b, the device exhibits a measurable photoresponse across the visible spectral range at zero bias, demonstrating a visible‐range photovoltaic response. Based on this spectral response, 510, 610, and 670 nm were selected as representative wavelengths for detailed power‐dependent I–t measurements.

**FIGURE 2:**
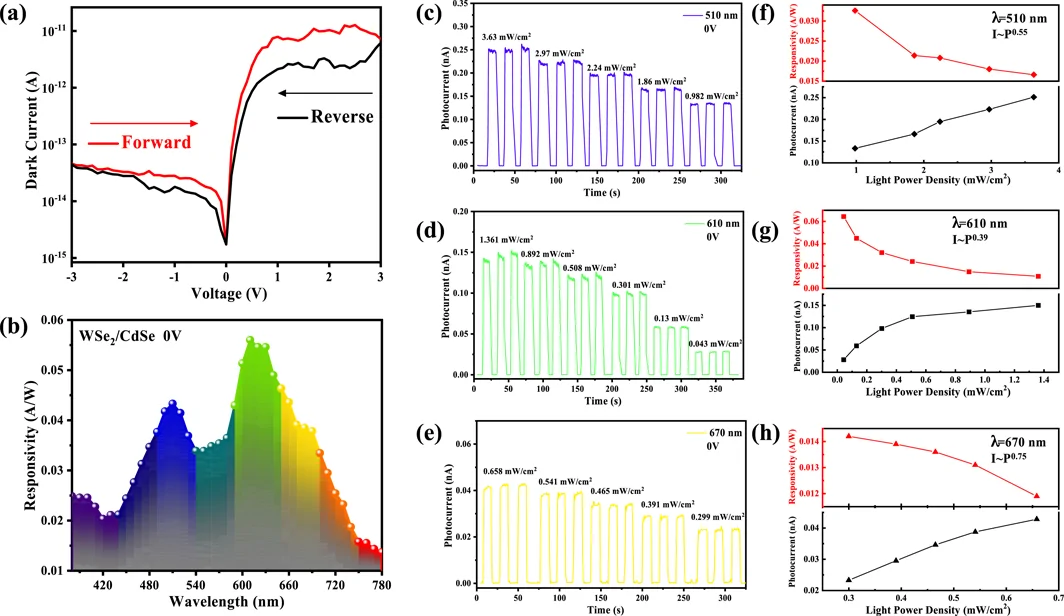
Zero‐bias self‐powered photoresponse of the WSe_2_/CdSe photodetector. (a) Dark I–V characteristics of the device. (b) Zero‐bias spectral responsivity used to identify representative wavelengths for subsequent power‐dependent measurements. (c–e) Power‐dependent I–t responses measured at 510, 610, and 670 nm, respectively, under different incident power densities at 0 V. (f–h) Power‐density‐dependent photocurrent, responsivity, and power‐law fitting extracted at 510, 610, and 670 nm, respectively.

Power‐dependent I–t measurements were then performed at these three wavelengths under different incident power densities at V_ds_ = 0 V. As shown in Figure 2c–e, the device exhibits clear and repeatable on/off switching at 510, 610, and 670 nm without external bias. The photocurrent decreases stepwise with decreasing incident power density, confirming that the zero‐bias output can be modulated by optical intensity. These switching measurements demonstrate a reproducible self‐powered photoresponse of the WSe_2_/CdSe heterojunction.

The photocurrent, responsivity, and power‐law behavior extracted from the power‐dependent I–t measurements are summarized in Figure 2f–h. For all three wavelengths, the photocurrent increases monotonically with increasing incident power density, whereas the responsivity gradually decreases. The relationship between photocurrent and power density follows a sublinear power law:

(1) $$I_{\text{ph}}\propto P^{\theta }$$

with fitted *θ* values of approximately 0.55, 0.39, and 0.75 for 510, 610, and 670 nm, respectively. This sublinear behavior suggests that the zero‐bias photoresponse is influenced by trap occupancy and carrier recombination processes [39]. The gradual decrease in *R* with increasing power density is consistent with the observed sublinear scaling of photocurrent and progressive saturation of photoactive states [40].

The variation in *θ* with wavelength indicates different power‐dependent photoresponse behaviors under different excitation conditions. The larger *θ* value at 670 nm corresponds to a more nearly proportional power dependence, whereas the smaller value at 610 nm reflects stronger sublinear behavior, likely arising from differences in optical absorption, interfacial carrier dynamics, and trap occupancy.

The power‐dependent responsivity was evaluated at each wavelength using the corresponding incident power density and an effective illuminated area of 1015.71 μm^2^. Definitions of *R*, *EQE*, and the estimated D*, together with the normalization procedure, are provided in the Supporting Information S1 [41, 42]. Overall, Figure 2 establishes the zero‐bias visible photoresponse of the WSe_2_/CdSe heterojunction and shows that its photocurrent output can be modulated by both wavelength and incident power density.

### 2.3 Photovoltaic Output and Bias‐Dependent Photoresponse

To characterize the photovoltaic nature of the zero‐bias response, illuminated I–V characteristics were measured under 610 nm irradiation with different incident power densities. As shown in Figure 3a, the photocurrent increases over the whole voltage range with increasing light intensity. Finite short‐circuit current (*I*_sc_) and open‐circuit voltage (*V*_oc_) are observed under illumination, establishing the photovoltaic operation of the heterojunction [43].

**FIGURE 3:**
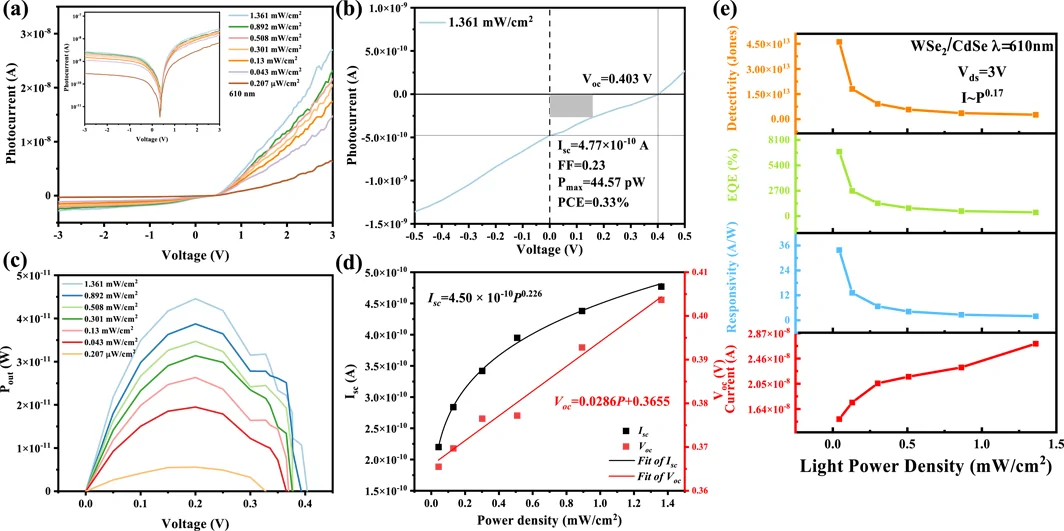
Photovoltaic output and bias‐dependent photoresponse of the WSe_2_/CdSe heterojunction. (a) Illuminated I–V curves measured under 610 nm light with different incident power densities; inset shows the corresponding low‐current region. (b) Representative photovoltaic output under 610 nm illumination, showing the extracted *V*_oc_, *I*_sc_, *FF*, *P*_max_, and *PCE*_app_. (c) Output *P*–*V* curves derived from the illuminated I–V characteristics under different incident power densities. (d) Power‐density‐dependent *I*_sc_ and *V*_oc_ extracted from the illuminated I–V curves under 610 nm illumination. The *I*_sc_ data are fitted using a power‐law function, while the *V*_oc_ data are fitted using an empirical linear function within the measured power‐density range. (e) Power‐density‐dependent photocurrent, *R*, *EQE*, and estimated D* measured at 610 nm under +3 V bias.

A representative self‐powered output is shown in Figure 3b. Under 610 nm illumination, the photovoltaic output increases with incident power density. At *P*_in_ = 1.361 mW/cm^2^, corresponding to an incident optical power of approximately 13.82 nW over the effective illuminated area of 1015.71 μm^2^, the device exhibits a *V*_*oc*_ of 0.403 V and an *I*_sc_ of 4.77 × 10^−10^ A. Using the same effective illuminated area, the corresponding zero‐bias responsivity is approximately 34.5 mA/W. The corresponding electrical output power (*P*_out_), fill factor (*FF*), and apparent power conversion efficiency (*PCE*_app_) were extracted from the illuminated I–V curves according to the definitions provided in the Supporting Information S1.

The photovoltaic output behavior is further supported by the power–voltage (P–V) analysis in Figure 3c. The P–V curves exhibit well‐defined maxima under different incident power densities, indicating finite electrical power output under illumination. These photovoltaic characteristics are consistent with interfacial carrier separation at the WSe_2_/CdSe interface.

The power‐dependent *I*_*sc*_ and *V*_*oc*_ were further extracted from the illuminated I–V curves and analyzed in Figure 3d. The *I*_*sc*_ increases monotonically with incident power density and follows a sublinear power‐law dependence of

(2) $$I_{sc}=4.50\times 10^{-10}P^{0.226}$$

where *P* is the incident power density in mW/cm^2^. The sublinear dependence indicates that the short‐circuit current is influenced by nonideal carrier generation, recombination, and trap‐related processes during zero‐bias photovoltaic operation. In contrast, *V*_*oc*_ increases gradually from 0.3655 to 0.4037 V and can be empirically fitted by

(3) $$V_{oc}=0.0286P+0.3655$$

within the measured power‐density range. The monotonic increase of *I*_*sc*_ and *V*_*oc*_ with incident power density further confirms the power‐dependent photovoltaic output of the WSe_2_/CdSe heterojunction.

The bias‐dependent photoresponse was then examined to evaluate carrier‐collection capability under an external electric field. As shown in Figure 3e, under 610 nm illumination and +3 V bias, the photocurrent increases monotonically with increasing incident power density. Meanwhile, the *R*, *EQE*, and estimated D* decrease overall as the optical excitation becomes stronger. This trend is consistent with sublinear photocurrent scaling and progressive saturation of photoactive states at higher excitation levels. The photocurrent under +3 V bias also follows a sublinear power‐law dependence, with a fitted exponent of approximately 0.17. Although the external bias enhances carrier drift and collection, the small power‐law exponent indicates strongly sublinear photocurrent scaling, likely reflecting the combined effects of trap occupancy, carrier recombination, and saturation processes [44, 45].

These results indicate that the WSe_2_/CdSe device operates in two related modes. At zero bias, the device operates through a junction photovoltaic response associated with the interfacial electrostatic asymmetry of the WSe_2_/CdSe heterojunction. Under external bias, the applied electric field further promotes carrier drift and collection, leading to enhanced photoresponse. Additional biased‐mode measurements under 460 nm illumination are provided in Supporting Information S1: Figure S3, where similar power‐dependent photocurrent enhancement and sublinear power‐law behavior are observed. These supplementary results further support the contribution of field‐assisted carrier extraction to the biased photoresponse [45].

### 2.4 Dynamic Photoresponse and Operational Stability

The dynamic photoresponse of the WSe_2_/CdSe heterojunction was investigated under 610 nm illumination. Figure 4a shows stable periodic switching at 100 Hz under 0, +3, and −3 V bias. At zero bias, the device maintains a clear self‐powered photocurrent modulation, whereas the modulation amplitude increases substantially under applied bias. The corresponding averaged single‐cycle responses are shown in Figure 4b. The rise and fall times were extracted using the 10%–90% and 90%–10% criteria, respectively. At 0 V, the device exhibits rise and fall times of approximately 1.09 and 0.99 ms. Under +3 V bias, these values decrease to approximately 165 and 191 μs, respectively, whereas under −3 V they are approximately 291 and 491 μs. The shorter switching times under both bias polarities demonstrate bias‐assisted acceleration of the electrical response, consistent with enhanced field‐assisted carrier collection. The difference between +3 and −3 V further reveals a polarity dependence in the switching dynamics. The frequency‐dependent response was further examined from 20 to 1000 Hz. As shown in Figure 4c, the relative modulation amplitude is defined as A_(f)_/A_ref_, where A_ref_ is the mean modulation amplitude at 20, 50, 100, and 200 Hz for each bias condition. At zero bias, the relative amplitude decreases to 45.9% at 500 Hz and 24.2% at 1000 Hz, whereas it remains at 101.5% and 96.5%, respectively, under +3 V bias. The inset of Figure 4c shows an averaged single‐cycle response from the 20‐cycle acquisition at +3 V and 1000 Hz, yielding rise and fall times of approximately 68 and 83 μs using the 10%–90% and 90%–10% criteria, respectively. The corresponding multi‐cycle I–t traces at 20, 50, 100, 200, 500, and 1000 Hz are provided in Supporting Information S1: Figure S5. These results demonstrate improved high‐frequency modulation retention under applied bias [45].

**FIGURE 4:**
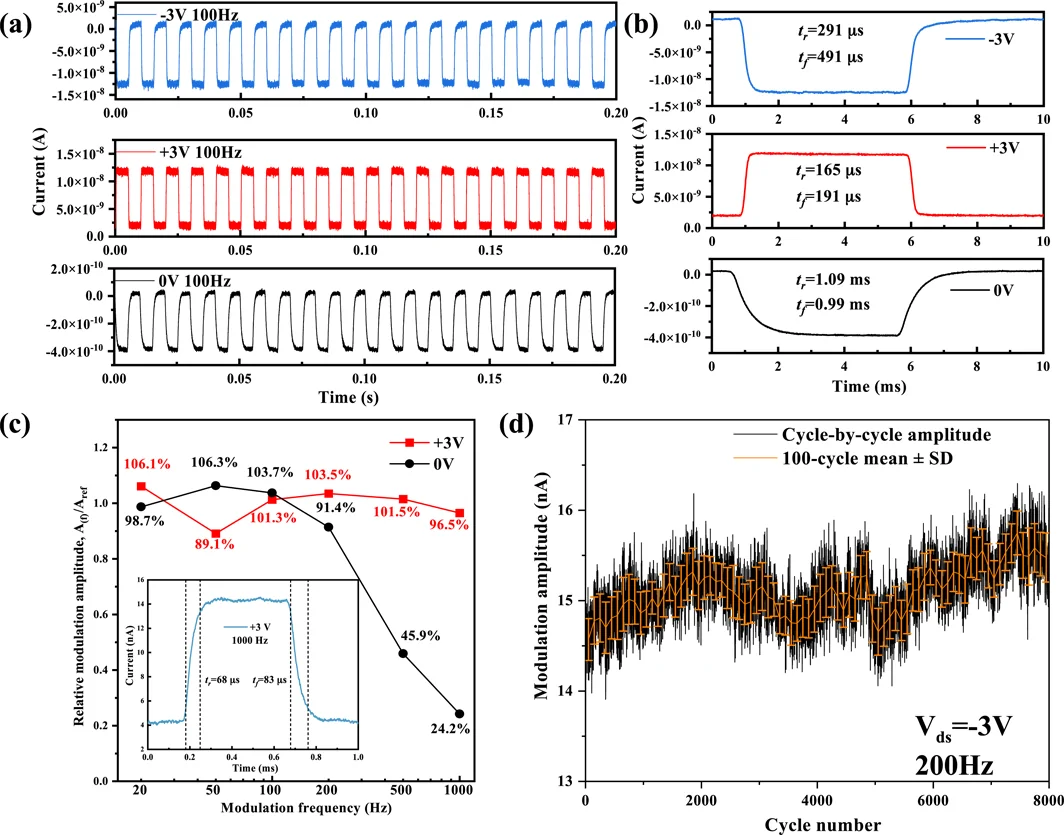
Dynamic photoresponse and operational stability of the WSe_2_/CdSe photodetector. (a) Periodic photocurrent switching measured under 610 nm illumination at 100 Hz under −3, +3, and 0 V bias. (b) Averaged single‐cycle responses and the corresponding rise (*t*_r_, 10%–90%) and fall (*t*_f_, 90%–10%) times under different bias conditions. (c) Relative modulation amplitude, A_(f)_/A_ref_, from 20 to 1000 Hz at 0 and +3 V. For each bias condition, A_ref_ is the mean amplitude at 20, 50, 100, and 200 Hz; the percentage labels indicate 100 × A_(f)_/A_ref_. Inset: averaged single‐cycle response from the 20‐cycle acquisition at +3 V and 1000 Hz, with rise/fall times of approximately 68/83 μs. (d) Repeated‐switching stability under −3 V bias and 200 Hz optical modulation over 8000 consecutive cycles. The black trace represents the cycle‐by‐cycle modulation amplitude, and the orange symbols represent the mean ± standard deviation calculated over successive 100‐cycle intervals.

The power‐dependent, quasi‐static, and transient measurements reveal complementary aspects of the photoresponse. The sublinear power dependence and I–V hysteresis suggest the participation of trap‐related charge processes, whereas the transient measurements show how applied bias accelerates electrical switching and improves high‐frequency modulation retention. Together, these results highlight the role of the applied field in regulating the dynamic photoresponse of the WSe_2_/CdSe heterojunction.

The repeated‐switching stability was evaluated under −3 V bias and 200 Hz optical modulation. Figure 4d shows the cycle‐by‐cycle modulation amplitude over 8000 consecutive switching cycles together with the mean ± standard deviation calculated over successive 100‐cycle intervals. The modulation amplitude remains stable throughout the measurement, with an overall average of approximately 15.11 ± 0.35 nA, demonstrating good repeatability under prolonged periodic excitation.

Additional electrical characteristics are summarized in Supporting Information S1: Figure S4. The asymmetry of dark current and the increasing rectification ratio with the absolute bias voltage confirm the diode‐like transport behavior associated with the WSe_2_/CdSe heterointerface and asymmetric contacts [46, 47, 48, 49]. Scan‐speed‐dependent I–V curves and forward/reverse sweep measurements reveal hysteresis, suggesting that trap‐mediated charge redistribution and dynamic barrier modulation participate in carrier transport [50, 51, 52]. For comparison, Supporting Information S1: Figure S4f summarizes the *V*_*oc*_ and *I*_*sc*_ of the present device together with representative self‐powered photodetectors reported in the literature [1, 5, 14, 15, 22, 24, 53]. Additional photoresponse metrics, including responsivity, detectivity, and response time, are summarized in Supporting Information S1: Table S1. Overall, the WSe_2_/CdSe heterojunction combines zero‐bias millisecond‐scale response, bias‐accelerated microsecond response, improved modulation‐amplitude retention up to 1 kHz, and repeatable switching over 8000 cycles.

### 2.5 Spatially Resolved Photoresponse and Interfacial Electronic Structure

To further clarify the spatial origin of the photovoltaic response, spatially resolved photocurrent mapping was performed on the WSe_2_/CdSe device under 610 nm illumination using a laser spot of approximately 1 μm and a scan step of 1 μm. Figure 5a shows the in situ optical image recorded during the mapping measurement, where the WSe_2_ nanosheet, CdSe nanobelt, In/Au electrodes, and WSe_2_/CdSe overlap region are clearly identified and outlined. The scale bar corresponds to 5 μm. The use of the in situ optical image allows a direct comparison between the device geometry and the spatial photocurrent distribution. As shown in Figure 5b, the strongest zero‐bias photocurrent is localized near the WSe_2_/CdSe overlap region. Quantitative region‐of‐interest analysis gives a background‐corrected photocurrent magnitude of 5.94 ± 4.75 nA in the overlap region, compared with 1.42 ± 2.37 nA in the WSe_2_‐only region, 0.20 ± 0.34 nA in the CdSe‐only region, and 0.119 ± 0.042 and 0.115 ± 0.054 nA at the two metal‐contact regions, respectively (Supporting Information S1: Figure S6). The mean overlap‐region response is approximately 50 times larger than the contact response, identifying the heterojunction region as the principal source of the zero‐bias photocurrent [54, 55]. The bias‐dependent spatial photoresponse was further examined under +3 V bias. As shown in Figure 5c, the photocurrent magnitude is significantly enhanced compared with that at 0 V, and the response extends along the carrier‐transport pathway between the two electrodes.

**FIGURE 5:**
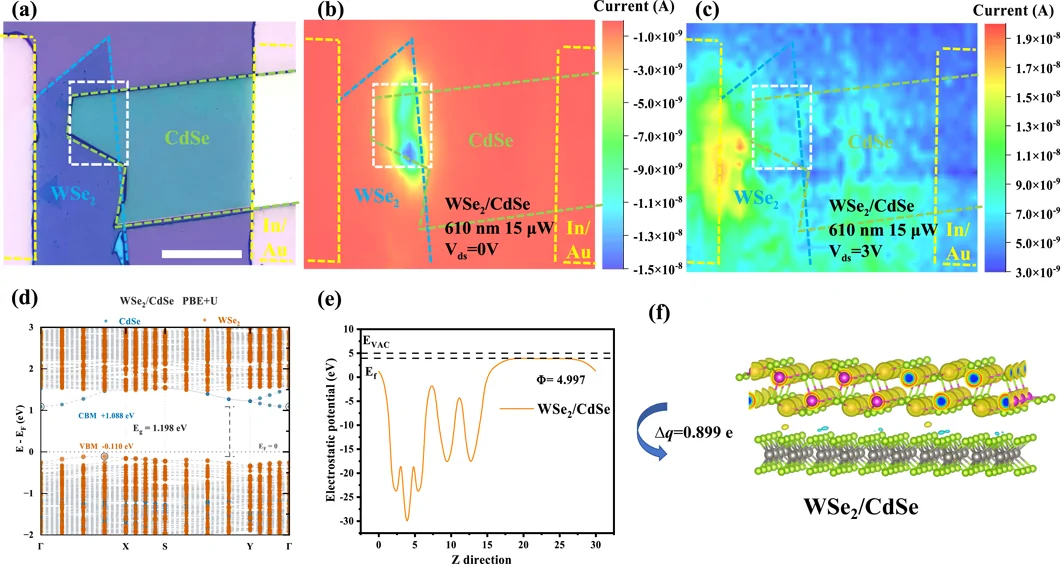
Spatially resolved photoresponse and interfacial electronic structure of the WSe_2_/CdSe heterojunction. (a) In situ optical image of the mapped WSe_2_/CdSe device, with WSe_2_, CdSe, In/Au electrodes, and the WSe_2_/CdSe overlap region outlined. Scale bar: 5 μm. (b) Spatially resolved photocurrent mapping measured under 610 nm illumination at 0 V. (c) Spatially resolved photocurrent mapping measured under 610 nm illumination at +3 V. The dashed outlines in panels (a–c) indicate the WSe_2_ nanosheet, CdSe nanobelt, electrodes, and overlap region. Photocurrent magnitudes are used for comparison; the current sign follows the source–drain measurement convention. (d) Material‐projected PBE+U band structure of the WSe_2_/CdSe heterojunction, showing a fundamental gap of approximately 1.20 eV. (e) Plane‐averaged electrostatic potential along the vertical direction, yielding a calculated work function of approximately 5.00 eV. (f) Differential charge‐density distribution of the WSe_2_/CdSe heterojunction, with a Bader charge‐transfer magnitude of approximately 0.90 e per simulation supercell. Yellow and cyan denote electron depletion and accumulation, respectively. The isosurface value is set to 0.0008 e Å^−3^.

Density functional theory (DFT) calculations were used to examine the band‐edge states and interfacial electronic redistribution of the WSe_2_/CdSe heterostructure. The PBE+U calculation yields a fundamental bandgap of approximately 1.20 eV. The material‐projected band structure in Figure 5d assigns the valence‐band maximum predominantly to WSe_2_ and the conduction‐band minimum predominantly to CdSe, supporting a type‐II band‐edge configuration. Figure 5e presents the plane‐averaged electrostatic potential along the stacking direction, from which a work function of approximately 4.997 eV is obtained.

The differential charge‐density distribution in Figure 5f shows electron accumulation and depletion concentrated near the interface. Bader analysis gives a net charge‐transfer magnitude of approximately 0.90 e per simulation supercell, quantifying the equilibrium electronic redistribution between the constituents. The material‐resolved band edges and interface‐localized charge redistribution provide a microscopic framework for interpreting the measured SKPM contrast and the overlap‐dominated zero‐bias photocurrent.

A BP/CdSe vertical heterojunction was examined as an auxiliary comparison, focusing on the interfacial electrostatic contrast and its associated zero‐bias photoresponse. Optical microscopy confirms a continuous overlap region, whereas Raman spectra collected before and after the electrical measurements retain the characteristic BP vibrational modes without pronounced peak broadening (Supporting Information S1: Figure S7). SKPM measurements yield a substantially smaller contact‐potential‐difference contrast of approximately 41 ± 9 mV across the BP/CdSe interface (Supporting Information S1: Figure S1), compared with 293 ± 15 mV for WSe_2_/CdSe. Correspondingly, the BP/CdSe device exhibits only weak zero‐bias photoresponse, whereas a more pronounced response emerges under applied bias (Supporting Information S1: Figure S8). The auxiliary DFT analysis in Supporting Information S1: Figure S9 illustrates electronic‐structure changes and interfacial charge redistribution in the BP/CdSe heterostructure. These observations reveal distinct interfacial electrostatic contrasts and zero‐bias photovoltaic responses in the two CdSe‐based heterojunctions.

The calculated band structures and work functions of isolated WSe_2_ and CdSe are shown in Supporting Information S1: Figure S10, whereas the corresponding TDOS and PDOS results are provided in Supporting Information S1: Figure S11 to illustrate the orbital contributions and interface‐induced electronic‐structure modulation.

Taken together, the spatial photocurrent mapping and electronic‐structure analysis establish a consistent picture in which the WSe_2_/CdSe overlap region dominates the zero‐bias photoresponse and exhibits pronounced interfacial electrostatic and charge redistribution. Under external bias, carrier collection extends over a broader portion of the device, accounting for the enhanced biased photoresponse.

### 2.6 Self‐Powered Optical Imaging and Key/Payload‐Assisted Optical Information Recognition

As shown in Figure 6a, a patterned optical input is projected onto the device and the corresponding photocurrent is collected pixel by pixel under zero‐bias operation. The reconstructed images of “Y” and “N” in Figure 6b,c exhibit clear contrast between illuminated and nonilluminated regions, demonstrating self‐powered visible‐light imaging [54].

**FIGURE 6:**
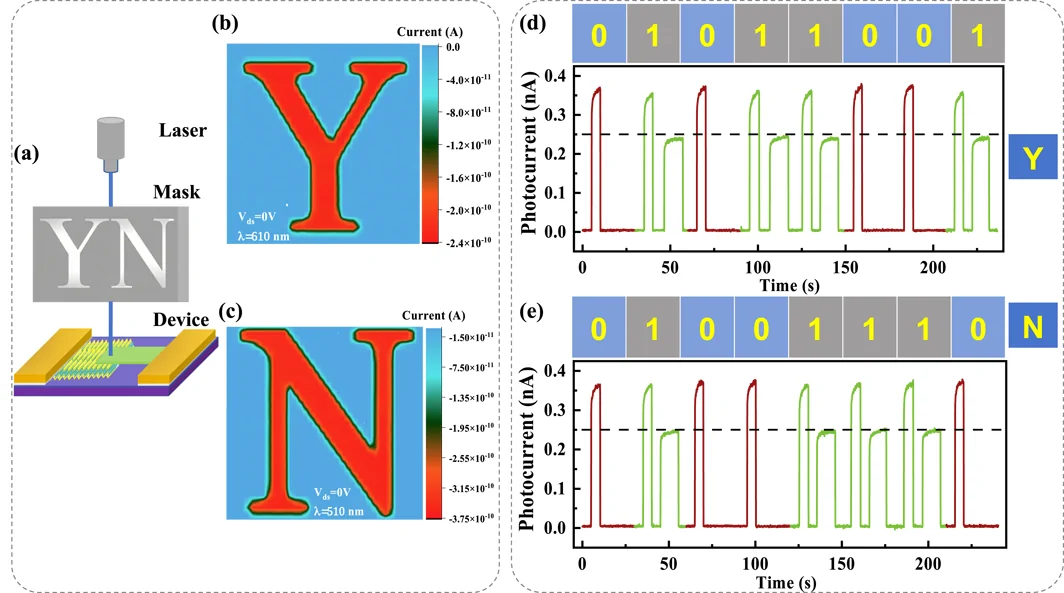
Self‐powered optical imaging and key/payload‐assisted optical information recognition using the WSe_2_/CdSe heterojunction. (a) Schematic of the self‐powered optical imaging process. (b, c) Reconstructed optical images of “Y” and “N”, respectively, under zero‐bias operation. (d, e) Zero‐bias photocurrent traces and corresponding 8‐bit decoding results for “Y” (01011001) and “N” (01001110), respectively.

In addition to imaging, the wavelength‐dependent zero‐bias photocurrent enables optical information recognition using combined wavelength and temporal encoding. As shown in Supporting Information S1: Figure S12a, 510 and 610 nm illumination generate distinguishable photocurrent levels of approximately 0.36 and 0.25 nA, respectively. In the decoding scheme, the 510 nm optical pulse serves as the key signal and is applied during the 5–10 s interval of each 30 s bit period. Binary “0” is assigned when the key pulse is present without a subsequent 610 nm payload pulse, whereas binary “1” is assigned when the key pulse is followed by a 610 nm payload pulse during the 15–25 s interval. Decoding combines the wavelength‐dependent photocurrent levels with the prescribed key and payload time windows. The detailed decoding process is provided in Supporting Information S1: Figure S12.

The feasibility of this decoding process was verified using an 8‐bit optical information recognition demonstration. As shown in Figure 6d,e, the sequential optical inputs are converted into the 8‐bit binary sequences 01011001 and 01001110 corresponding to “Y” and “N”, respectively.

The repeatability and recognition accuracy of the decoding scheme were further evaluated using multiple 8‐bit character sequences. The characters “Y” (01011001) and “N” (01001110) were each tested in three independent trials, whereas “G”, “K”, “M”, “S”, “U”, and “Z” were additionally evaluated as representative 8‐bit sequences (Supporting Information S1: Figures S13 and S14). All tested sequences were correctly decoded, corresponding to 12 successful character‐recognition trials and 96 correctly identified bits, with a character‐recognition accuracy of 100% and no bit errors among the 96 tested bits (BER = 0 under the tested conditions).

To further examine the selectivity of the key/payload‐assisted decoding rule, incorrect optical input combinations were also evaluated. As summarized in Supporting Information S1: Figure S15, 15 invalid key/payload combinations failed to reproduce the target decoding output because at least one of the predefined criteria involving optical input identity, photocurrent level, or pulse duration was not satisfied. These results demonstrate the selectivity of the decoding process to the combined wavelength‐, amplitude‐, and time‐window‐dependent input conditions.

Overall, the imaging and optical recognition demonstrations show that the WSe_2_/CdSe heterojunction can convert visible optical inputs into spatially and temporally distinguishable electrical outputs under zero‐bias operation.

## 3 Conclusions

In summary, we investigated the photovoltaic behavior and optical information recognition capabilities of a mixed‐dimensional WSe_2_/CdSe heterojunction. The spatially resolved photocurrent measurements demonstrate that the semiconductor overlap region contributes substantially more strongly to the zero‐bias photoresponse than the metal‐contact regions. Together with the reproducible SKPM contrast and the material‐projected electronic‐structure calculations, these observations support a physical picture in which the heterointerface plays an important role in photovoltaic carrier separation. The BP/CdSe comparison reveals a smaller interfacial potential contrast and a weaker zero‐bias photoresponse, further highlighting the dependence of photovoltaic behavior on the interfacial configuration.

The dynamic measurements reveal distinct zero‐bias and bias‐assisted operating characteristics. The device exhibits millisecond‐scale switching at zero bias, whereas applied bias accelerates the measured response and improves modulation‐amplitude retention up to 1 kHz.

Beyond photodetection, the heterojunction enables visible‐light imaging and wavelength‐ and time‐window‐dependent optical information recognition without external bias. The successful recognition of all 96 tested bits illustrates the feasibility of combining self‐powered optical sensing with predefined information‐decoding functions. This work thus extends the functionality of WSe_2_/CdSe mixed‐dimensional heterojunctions from photoconductive detection toward zero‐bias photovoltaic sensing and optical information recognition.

## 4 Experimental Section

### 4.1 Materials and Heterojunction Assembly

WSe_2_ nanosheets were prepared by chemical vapor deposition, following a previously reported procedure [56]. The vertical WSe_2_/CdSe vdWH was assembled by transferring a CdSe nanobelt onto the WSe_2_ nanosheet to form a well‐defined overlap junction region between two electrodes. The WSe_2_/CdSe overlap region served as the active heterojunction region for zero‐bias photovoltaic response and bias‐assisted photodetection measurements.

A vertical BP/CdSe device was also fabricated as an auxiliary CdSe‐based control for comparison of the zero‐bias photoresponse.

### 4.2 Characterization and Device Measurements

Optical microscopy (OM), Raman spectroscopy, steady‐state photoluminescence (PL), and time‐resolved photoluminescence (TRPL) measurements were performed using a confocal Raman system (WITec α‐300R). Raman and steady‐state PL spectra were collected under 532 nm laser excitation, whereas TRPL measurements were performed under 375 nm pulsed laser excitation. The thicknesses of the flakes were characterized by atomic force microscopy (AFM), and the surface‐potential distribution was measured by scanning Kelvin probe microscopy (SKPM) on an Asylum Cypher S platform. The crystalline structures of the as‐prepared WSe_2_ and CdSe were further examined by TEM, HRTEM, and SAED using an FEI Talos F200S.

Electrical characterization was performed with a semiconductor parameter analyzer (Keithley 4200‐SCS) at room temperature. A monochromator (Newport CS260) and a xenon lamp were used as a wide‐range monochromatic light source for photodetection measurements. Photocurrent mapping and optical imaging were performed using a scanning photoelectric test system (Metatest MStarter 200). Transient photoresponse measurements were performed under 610 nm illumination. For comparison among 0, +3, and −3 V, the rise and fall times were extracted from averaged single‐cycle waveforms at 100 Hz using the 10%–90% and 90%–10% criteria, respectively. The same criteria were applied to the averaged single‐cycle response from the 20‐cycle acquisition at +3 V and 1000 Hz. Frequency‐dependent I–t measurements were carried out at 20, 50, 100, 200, 500, and 1000 Hz under 0 and +3 V bias. Repeated‐switching stability was evaluated under −3 V bias and 200 Hz optical modulation over 8000 consecutive cycles. For spatial photocurrent mapping, a laser spot of approximately 1 μm and a scan step of 1 μm were used. The electrical signals were sampled at 100 kSa/s for the zero‐bias transient measurements and 1 MSa/s for the biased measurements, with digital filtering disabled. The reported rise and fall times characterize the electrical response of the device–measurement system under the specified conditions. For key/payload‐assisted optical information recognition, optical pulse sequences were applied to the device, and the output photocurrent states were decoded into binary “0” and “1” according to the preset wavelength‐, photocurrent‐amplitude‐, and time‐window‐dependent decoding rule described in Supporting Information S1: Figure S12.

### 4.3 First‐Principles Calculation

First‐principles calculations were performed within the framework of density functional theory (DFT) using the Vienna Ab initio Simulation Package (VASP) [57]. The exchange–correlation interaction was described by the generalized gradient approximation (GGA) with the Perdew–Burke–Ernzerhof (PBE) functional [58]. To improve the description of the Cd‐derived electronic states, an on‐site Hubbard correction was introduced within the DFT+U approach with an effective *U*_*eff*_ = 7.5 eV applied to the Cd 4d orbitals. A vacuum layer of 20 Å was introduced along the out‐of‐plane direction to avoid spurious interactions between periodic images. The plane‐wave cutoff energy was set to 400 eV, and the Brillouin zone was sampled using a 2 × 1 × 1 Monkhorst‐Pack k‐point mesh for structural optimization and electronic‐structure calculations. The atomic positions were fully relaxed until the residual force on each atom was less than 0.05 eV/Å and the total‐energy convergence criterion reached 1.0 × 10^−5^ eV. Van der Waals interaction was taken into account using the DFT‐D3 correction scheme [59].

Based on the optimized structures, the band structures and work functions referenced to the vacuum level of pristine WSe_2_, pristine CdSe, pristine BP, WSe_2_/CdSe, and BP/CdSe were obtained. The total and partial densities of states (TDOS/PDOS) of the pristine components and heterojunctions were further analyzed. Differential charge‐density and Bader charge analyses were performed to evaluate interfacial charge redistribution and net charge transfer after heterojunction formation. The differential charge density was calculated as follows: $\Delta \rho =\rho_{\text{heterojunction}}-\rho_{\text{component}1}-\rho_{\text{component}2}$, where *ρ*_heterojunction_, *ρ*_component1_, and *ρ*_component2_ represent the charge densities of the heterostructure and the corresponding isolated components calculated in the same atomic configuration. The PBE+U calculations were used to analyze the electronic structure, electrostatic potential, density of states, and interfacial charge redistribution of the CdSe‐containing heterostructures.

## Author Contributions

**Lei Liu:** data curation, writing – original draft, writing – review and editing, investigation, conceptualization, formal analysis, visualization, validation. **Haoyun Dou:** software, formal analysis. **Haiqiang Huang:** methodology, conceptualization, visualization, validation. **Fan Mu:** methodology. **Yan Yang:** methodology. **Hui Zhang:** conceptualization. **Feng Song:** supervision. **Yingkai Liu:** supervision, writing – review and editing, funding acquisition, resources, project administration.

## Funding

This work is funded by the National Natural Science Foundation of China (Grants 12264056 and 62164013), Key Applied Basic Research Program of Science and Technology Commission Foundation of Yunnan Province (Grant 202501AS070001), and Yunnan Expert Workstation Project (Grant 202205AF150008).

## Conflicts of Interest

The authors declare no conflicts of interest.

## Supporting information


Supporting Information S1

## Data Availability

The data that support the findings of this study are available within the article and its Supporting Information. Additional data are available from the corresponding author upon reasonable request.
