# Supplementary material for: Self‐Powered Photodetection and Optical Information Recognition in a Mixed‐Dimensional WSe2/CdSe Heterojunction
Source: Nanophotonics. 2026 Nov 7;15(21):e70362. doi: 10.1002/nap2.70362 (PMC13743990; doi:10.1002/nap2.70362)
Supplement: Supplementary file 1 [file NAP2-15-e70362-s001.docx]

Supporting Information

Self-Powered Photodetection and Optical Information Recognition in a Mixed-Dimensional WSe_2_/CdSe Heterojunction

Lei Liu^1,2,3^, Haoyun Dou^1,2^, Haiqiang Huang^1,2,3^, Fan Mu^1,2^, Yan Yang^1,2,3^, Hui Zhang^1,2^, Feng Song^3,4^, Yingkai Liu^1,2,3,*^

**S1. Repeated SKPM analysis of the WSe_2_/CdSe and BP/CdSe interfaces**

Repeated scanning Kelvin probe microscopy (SKPM) line profiles were acquired across the WSe_2_/CdSe and BP/CdSe interfaces to evaluate the reproducibility of the interfacial contact-potential-difference contrast. For WSe_2_/CdSe, three independent profiles yield ΔV_CPD_ values of approximately 281, 287, and 309 mV, corresponding to an average of 293 ± 15 mV. For BP/CdSe, the corresponding values are approximately 34.7, 51.3, and 37.5 mV, giving an average of 41 ± 9 mV.

**Figure S1.** Repeated SKPM analysis of the WSe_2_/CdSe and BP/CdSe interfaces. (a, c) Surface-potential map and three independent line profiles across the WSe_2_/CdSe interface. (b, d) Corresponding measurements across the BP/CdSe interface. The average contact-potential-difference contrasts are 293 ± 15 mV for WSe_2_/CdSe and 41 ± 9 mV for BP/CdSe.

**S2. TEM, HRTEM and SAED characterizations of WSe_2_ and CdSe**

The crystalline structures of the constituent WSe_2_ nanosheet and CdSe nanobelt were examined before heterojunction assembly. The clear lattice fringes and well-defined diffraction spots confirm the good crystallinity of both materials.

**Figure S2.** TEM, HRTEM and SAED characterizations of WSe_2_ and CdSe. (a, d) Low-magnification TEM images of WSe_2_ and CdSe, respectively. (b, e) HRTEM images showing the corresponding lattice fringes. (c, f) SAED patterns confirming the crystalline structures of WSe_2_ and CdSe.

**Calculation of photodetection parameters and area normalization**

The photoresponse parameters of the WSe_2_/CdSe photodetector were calculated from the measured photocurrent under specified illumination and bias conditions. The responsivity (*R*), external quantum efficiency (*EQE*) and the specific detectivity (*D**) are calculated using the following equations:

$\text{R}\text{=}\frac{\text{I}_{\text{lig}\text{h}\text{t}}\text{−}\text{I}_{\text{dark}}}{\text{P}_{\text{in}}\text{×}\text{A}_{eff}}$	(1)

$\text{EQE}\text{=}\frac{\text{h}\text{×}\text{c}\text{×}\text{R}}{\text{e}\text{×}\text{λ}}\text{×100\%}$	(2)

$\text{D}^{\text{∗}}\text{=}\frac{\text{R}\text{×}\sqrt{\text{A}_{eff}}}{\sqrt{\text{2}\text{e}\text{×}\text{|}\text{I}_{\text{dark}}|}}$	(3)

Here, *I_light_* and *I_dark_* are the currents measured under illumination and in the dark, respectively, and *I_ph_*=*I_light_*-*I_dark_*. *P_in_* is the incident power density, and *A_eff_* is the effective illuminated area. The symbols *h*, *e*, *c*, and *λ* denote Planck’s constant, the elementary charge, the speed of light, and the illumination wavelength, respectively.^[41,42]^ Area-normalized photoresponse parameters were calculated using A_eff_=1015.71 μm^2^. The geometrical WSe_2_/CdSe overlap area is 161.95 μm^2^. This normalization provides a common basis for evaluating the power-dependent photoresponse under the stated illumination conditions. The reported D* values are shot-noise-limited estimates calculated from the measured dark-current baseline. At zero bias, this baseline approaches the instrumental detection floor. For photovoltaic output analysis, the electrical output power (*P_out_*), fill factor (*FF*), and apparent power conversion efficiency (*PCE_app_*) were calculated as follows:

$\text{P}_{\text{out}}\text{=}\text{-}\text{I}_{\text{ds}}\text{×}\text{V}_{\text{ds}}$	(4)

$\text{FF}\text{=}\frac{\text{P}_{\text{out}\text{,}\text{max}}}{|\text{I}_{\text{sc}}\text{|}\text{×}\text{V}_{\text{oc}}}$	(5)

$\text{PCE}_{app}\text{=}\frac{\text{P}_{\text{out}\text{,}\text{max}}}{\text{P}_{\text{in}}A_{eff}}$	(6)

where *P_out,max_* is the maximum electrical output power extracted from the illuminated I-V curve, and *A_eff_*=1015.71 μm^2^ is the effective illuminated area used for the area-normalized analysis. The apparent monochromatic power conversion efficiency, *PCE_app_*, quantifies the maximum electrical output relative to the incident optical power over *A_eff_* under the specified illumination conditions.

**S3. Additional photovoltaic and bias-assisted photoresponse under 460 nm illumination**

The wavelength dependence of bias-assisted photodetection was further examined under 460 nm illumination. As shown in **Figure S3(a)**, the WSe_2_/CdSe heterojunction exhibits a visible spectral response under +3 V bias. The illuminated I-V curves in **Figure S3(b)** show that the current increases with increasing incident power density, which is consistent with bias-assisted carrier extraction. A representative photovoltaic-output extraction under 460 nm illumination is shown in **Figure S3(c)**. The power-dependent photocurrent, *R*, *EQE*, and *D** summarized in **Figure S3(d)** further indicate that the biased photoresponse can be modulated by incident optical power. These results complement the 610 nm measurements in the main text and support the bias-modulated photodetection behavior of the WSe_2_/CdSe heterojunction.

**Figure S3.** Additional photovoltaic and bias-assisted photoresponse under 460 nm illumination. (a) Responsivity spectrum measured under +3 V bias. (b) Illuminated I-V curves measured under 460 nm light with different incident power densities. (c) Representative photovoltaic-output extraction from the illuminated I-V curve under 460 nm illumination. (d) Power-density-dependent photocurrent, responsivity, EQE, and estimated detectivity measured under 460 nm illumination at +3 V.

**S4. Additional electrical characteristics and operational stability of the WSe_2_/CdSe photodetector**

Additional electrical measurements were performed to clarify the transport behavior and operational robustness of the WSe_2_/CdSe device. As shown in **Figure S4(a)**, the dark I-V curve exhibits asymmetric transport behavior, consistent with the rectifying characteristics of the vertical heterojunction. The voltage-dependent rectification ratio extracted in **Figure S4(b)** further supports diode-like transport associated with the WSe_2_/CdSe heterointerface and asymmetric contacts.

The long-term storage stability of the device was evaluated by measuring the photoresponse after different storage periods. **Figure S4(c)** compares the photoresponse measured initially and after 120 and 360 days of storage, showing that a measurable photoresponse is retained.

Scan-speed-dependent I–V curves and forward/reverse sweep measurements were performed to examine quasi-static transport. The sweep-dependent hysteresis in **Figures S4(d,e)** indicates that charge redistribution contributes to the transport behavior.

The *V_oc_* and *I_sc_* values of the present WSe_2_/CdSe device are compared with representative self-powered photodetectors in **Figure S4(f)**.^[1,5,14,15,22,24,53]^ In addition, **Figure S4(g)** presents illuminated I–V characteristics of four additional WSe_2_/CdSe devices. These devices exhibit photovoltaic outputs with device-dependent V_OC_ and I_SC_ values.

**Figure S4.** Additional electrical characteristics and long-term stability of the WSe_2_/CdSe photodetector. (a) Dark I-V characteristics. (b) Rectification ratio extracted at different bias voltages. (c) Long-term storage stability. (d) Scan-speed-dependent I-V characteristics. (e) Forward and reverse dark I-V sweeps. (f) Comparison of *V_oc_* and *I_sc_* with representative self-powered photodetectors reported in the literature. (g) Illuminated I–V characteristics of four additional WSe_2_/CdSe devices.

**S5. Frequency-dependent temporal photoresponse of the WSe_2_/CdSe photodetector**

Frequency-dependent I-t measurements were performed under 610 nm illumination from 20 to 1000 Hz at 0 and +3 V. At zero bias, the modulation amplitude becomes progressively attenuated at higher frequencies. Under +3 V bias, distinct periodic switching is retained throughout the measured range, consistent with the relative modulation amplitude summarized in **Figure 4(c)** of the main text.

**Figure S5.** Frequency-dependent temporal photoresponse of the WSe_2_/CdSe photodetector. I–t curves measured under 610 nm illumination at modulation frequencies of 20, 50, 100, 200, 500, and 1000 Hz under (a) 0 V and (b) +3 V bias. The zero-bias modulation amplitude decreases markedly at high frequencies, whereas pronounced periodic switching is retained under +3 V over the measured frequency range.

**S6. Quantitative region-of-interest analysis of photocurrent mapping**

Region-of-interest (ROI) statistics were extracted from the spatial photocurrent maps using morphology-defined regions for the WSe_2_/CdSe overlap, WSe_2_-only, CdSe-only, two metal-contact regions, and the background. To minimize boundary mixing, each ROI was restricted to points at least one scan step (~1 μm) inside the morphological boundary. The background-corrected magnitude was calculated relative to the median background level. At 0 V, the overlap region gives 5.94 ± 4.75 nA, compared with 1.42 ± 2.37 nA for WSe_2_-only, 0.20 ± 0.34 nA for CdSe-only, 0.119 ± 0.042 nA for Contact 1, 0.115 ± 0.054 nA for Contact 2, and 0.090 ± 0.084 nA for the background. Under +3 V, the response becomes spatially broader and larger contributions are observed outside the overlap region.

**Figure S6.** Quantitative ROI analysis of spatial photocurrent mapping. (a) Morphology-defined regions used for statistical analysis, including the WSe_2_/CdSe overlap, WSe_2_-only, CdSe-only, Contact 1, Contact 2, and background regions. (b,c) Background-corrected photocurrent magnitude obtained from the defined ROIs at 0 and +3 V, respectively. Error bars represent the standard deviation of the spatial sampling points within each ROI.

**S7. Structural and Raman characterization of the BP/CdSe control device**

The BP/CdSe control device was examined before and after the electrical measurements to assess the integrity of the BP flake and the overlap region. Optical images show that the device morphology remains intact. Raman spectra collected at two BP positions and the Au/BP region retain the characteristic BP modes after testing, with only minor peak shifts and no pronounced broadening.

**Figure S7.** Structural characterization of the BP/CdSe control device. (a, b) Optical images recorded before and after the electrical measurements. (c, d) Raman spectra collected at representative BP and Au/BP positions before and after testing.

**S8. Electrical photoresponse of the BP/CdSe control device**

A vertical BP/CdSe device was used as an auxiliary CdSe-based control. The device exhibits only weak and wavelength-limited photocurrent at zero bias, whereas a more pronounced response appears under applied bias. Together with the smaller SKPM potential contrast, these measurements provide a comparison of the interfacial electrostatics and photovoltaic behavior of the two CdSe-based heterojunctions.

**Figure S8.** Electrical photoresponse of the BP/CdSe control device. (a) Optical image of the BP/CdSe vdWH device. (b) Zero-bias wavelength-dependent photocurrent responses of the BP/CdSe device. (c) Bias-assisted photoresponse of the BP/CdSe device, showing increased photocurrent under applied bias compared with zero-bias operation.

**S9. Electronic-structure analysis of BP/CdSe**

DFT calculations were performed on BP/CdSe as an auxiliary system to examine the electronic-structure changes associated with vertical heterojunction formation. **Figures S9(a,b)** show the calculated band structure and plane-averaged electrostatic potential of isolated BP, while **Figures S9(c,d)** present the corresponding results for the BP/CdSe heterostructure. The calculated electronic structure and electrostatic potential are modified after vertical stacking.

The differential charge-density distribution in **Figure S9(e)** shows electron accumulation and depletion associated with interfacial charge redistribution. Bader analysis yields a net charge-transfer magnitude of approximately 0.052 e for the BP/CdSe simulation supercell. The BP/CdSe calculations provide an auxiliary description of electronic redistribution in the control heterojunction, complementing the experimental SKPM and zero-bias photoresponse measurements.

**Figure S9.** Auxiliary electronic-structure analysis of BP/CdSe. (a) Calculated band structure of isolated BP. (b) Plane-averaged electrostatic potential profile and work function of isolated BP. (c) Calculated band structure of the BP/CdSe heterostructure. (d) Plane-averaged electrostatic potential profile and work function of BP/CdSe. (e) Differential charge-density distribution of BP/CdSe, with a Bader charge-transfer magnitude of approximately 0.052 e per simulation supercell.

**S10. Calculated band structures and work functions of isolated WSe_2_ and CdSe**

The electronic structures of isolated WSe_2_ and CdSe were calculated using constituent structural models consistent with those used in the WSe_2_/CdSe heterojunction model. The PBE calculation yields a fundamental bandgap of approximately 1.519 eV for WSe_2_, while the PBE+U calculation gives a bandgap of approximately 1.616 eV for CdSe, with an effective Hubbard correction of *U_eff_* = 7.5 eV applied to the Cd 4d orbitals. The corresponding band structures are shown in **Figures S10(a,b)**.

The plane-averaged electrostatic potential profiles in **Figures S10(c,d)** yield work functions of approximately 4.715 and 5.535 eV for isolated WSe_2_ and CdSe, respectively. These results provide electronic-structure and work-function references for the constituent models used in the heterojunction analysis.

**Figure S10.** Calculated electronic structures and work functions of isolated WSe_2_ and CdSe. (a) PBE band structure of WSe_2_ with a fundamental bandgap of approximately 1.519 eV. (b) PBE+U band structure of CdSe with a fundamental bandgap of approximately 1.616 eV. (c,d) Corresponding plane-averaged electrostatic potential profiles, yielding work functions of approximately 4.715 and 5.535 eV for WSe_2_ and CdSe, respectively.

**S11. TDOS and PDOS analyses of WSe_2_, CdSe, WSe_2_/CdSe, and BP/CdSe**

The total and partial densities of states (TDOS and PDOS) were analyzed to examine the electronic-state contributions of the constituent materials and their heterostructures. **Figures S11(a,b)** show the TDOS and PDOS of isolated WSe_2_ and CdSe, respectively, providing references for the corresponding electronic structures.

**Figure S11(c)** presents the TDOS and PDOS of the WSe_2_/CdSe heterostructure. The redistribution of electronic states after vertical stacking reflects the modification of the electronic structure associated with heterojunction formation. The material-projected PBE+U band structure in **Figure 5(d)** further identifies the valence-band maximum as predominantly WSe_2_-derived and the conduction-band minimum as mainly CdSe-derived, supporting the proposed type-II band-edge configuration.

For comparison, **Figure S11(d)** presents the TDOS and PDOS of the auxiliary BP/CdSe heterostructure. The different electronic-state distributions illustrate the dependence of the calculated electronic structure on the constituent materials and interface configuration. The DOS results complement the band-structure and charge-density analyses by identifying the electronic-state contributions of the constituent materials.

**Figure S11.** Total and partial density-of-states analyses of isolated WSe_2_, isolated CdSe, WSe_2_/CdSe, and BP/CdSe. (a,b) TDOS and PDOS of isolated WSe_2_ and CdSe, respectively. (c) TDOS and PDOS of the WSe_2_/CdSe heterostructure, showing the contributions of different constituent orbitals to its electronic states. (d) TDOS and PDOS of the auxiliary BP/CdSe heterostructure. The calculations illustrate electronic-structure changes associated with heterojunction formation.

**S12. Wavelength- and time-window-dependent key/payload optical decoding process**

A key/payload-assisted optical decoding scheme was designed based on the wavelength-dependent zero-bias photocurrent of the WSe_2_/CdSe photodetector. As shown in **Figure S12(a)**, 510 and 610 nm illumination produce distinguishable photocurrent levels of approximately 0.36 and 0.25 nA, respectively. The 510 nm optical pulse is defined as the key signal, whereas the 610 nm pulse serves as the payload signal.

Each binary state is encoded within a 30 s time window using both wavelength and illumination timing. As illustrated in **Figure S12(b)**, the key pulse is applied during 5–10 s. When only the 510 nm key pulse is present and no payload pulse appears in the subsequent 15–25 s interval, the optical input is decoded as binary “0”. For binary “1”, the 510 nm key pulse during 5–10 s is followed by a 610 nm payload pulse during 15–25 s. The corresponding photocurrent traces therefore contain both wavelength-dependent amplitude and time-window information, as shown in **Figure S12(c)**.

This combined wavelength–amplitude–temporal encoding rule is used for the 8-bit optical information recognition demonstrated in Figure 6 and Figures S13–S15.

**Figure S12.** Wavelength- and time-window-dependent key/payload optical decoding process. (a) Zero-bias photocurrent responses generated by the 510 nm key and 610 nm payload signals, with characteristic photocurrent levels of approximately 0.36 and 0.25 nA, respectively. (b) Temporal encoding rule within each 30 s bit period. Binary “0” consists of a 510 nm key pulse applied during 5–10 s without a subsequent payload signal, whereas binary “1” contains the same key pulse followed by a 610 nm payload pulse during 15–25 s. (c) Representative photocurrent outputs corresponding to the binary “0” and “1” states.

**S13. Repeatability of 8-bit optical information recognition**

The repeatability of the 8-bit decoding process was evaluated using the characters “Y” (01011001) and “N” (01001110). Each character was measured in three independent trials, and all six character-recognition trials were correctly decoded using the same preset threshold and temporal rule.

**Figure S13.** Repeatability of 8-bit optical information recognition. Three independent decoding trials for (a) “Y” (01011001) and (b) “N” (01001110) under zero-bias operation. The dashed line indicates the preset photocurrent threshold used for distinguishing the two signal levels.

**S14. Extended 8-bit optical information recognition**

Additional 8-bit sequences corresponding to “G”, “K”, “M”, “S”, “U”, and “Z” were measured to evaluate the applicability of the decoding rule to different character patterns. All six sequences were correctly identified under the tested conditions.

**Figure S14.** Extended 8-bit optical information recognition using the WSe_2_/CdSe photodetector. Experimentally recorded photocurrent sequences and corresponding binary codes for “G” (01000111), “K” (01001011), “M” (01001101), “S” (01010011), “U” (01010101), and “Z” (01011010).

**S15. Invalid decoding outputs under incorrect key/payload combinations**

The invalid decoding outputs obtained under incorrect key/payload combinations are summarized in **Figure S15**. Valid decoding requires the predefined criteria for optical input identity, photocurrent level, and pulse timing to be satisfied. The 15 incorrect combinations fail to reproduce the target decoding output, showing the input selectivity of the key/payload-assisted rule. These results complement the 8-bit recognition results in Figure 6 and support the feasibility of self-powered optical information recognition and key/payload-assisted selective decoding.

**Figure S15.** Invalid decoding outputs under incorrect key/payload combinations. Fifteen invalid optical input combinations are compared with a valid reference output (red trace). The invalid combinations fail to satisfy the predefined wavelength-, photocurrent-level-, or temporal-window criteria and therefore do not reproduce the target decoding output.

**Table S1. Comparison of representative low-dimensional photodetectors**

| **Device** | **P_in_** | **λ (nm)** | **Responsivity** | **Detectivity**  **(Jones)** | **Rise/Fall Time** | **Ref.** |
| --- | --- | --- | --- | --- | --- | --- |
| MoS_2_/WS_2_ | 28.64 mW/cm^2^ | 532 | 4.36 mA/W | 4.36 × 10^13^ | ~ 4 ms | 1 |
| Bi_2_O_2_Se/InSe | 0.25 mW/cm^2^ | 350 | 0.47 A/W | 3.21 × 10^12^ | 48.5/41.7 μs ^a)^ | 5 |
| MSM WSe_2_ | 0.0537 mW/cm^2^ | 650 | 2.31 A/W | 9.16 × 10^11^ | NA | 14 |
| ITO/WSe_2_/SnSe_2_ | 0.267 mW/cm^2^ | 785 | 16.45 A/W | ~1 × 10^13^ | 8/32 μs | 15 |
| WSe_2_/Bi_2_Te_3_ | 0.01 mW/cm^2^ | 633 | 20.5 A/W | NA | 180/210 μs | 22 |
| PtSe_2_/GaAs | 0-49 mW/cm^2 b)^ | 850 | 67.2 mA/W | 6.7 × 10^12^ | 164/198 μs | 24 |
| ReSe_2_/PtSe_2_ | 7.29 mW/cm^2^ | 635 | 153 mA/W | 7.72 × 10^11^ | 811/720 ms | 53 |
| WSe_2_/CdSe | 0.043 mW/cm^2^  (V_ds_=0V) | 610 | 64.4 mA/W^d)^ | 5.26 × 10^12^ | 1.09/0.99 ms  (100 Hz) | This Work |
|  | 0.043 mW/cm^2^  (V_ds_=+3V)^c)^ | 610 | 33.8 A/W | 4.62 × 10^13^ | 165/191 μs  (100 Hz) |  |
| ^a)^ The rise/fall time of Bi_2_O_2_Se/InSe was measured at P_in_=15 mW/cm^2^.  ^b)^ The responsivity and detectivity are the maximum values reported at low incident power densities within the tested range of 0-49 mW/cm^2^.  ^c)^ The +3 V result of WSe_2_/CdSe corresponds to the bias-assisted photodetection mode of this work.  ^d)^ The responsivity values listed for WSe_2_/CdSe were obtained at an incident power density of 0.043 mW/cm^2^, whereas the representative zero-bias responsivity of 34.5 mA/W in the main text was calculated at 1.361 mW/cm^2^. | | | | | | |
